# Developments in Plant Proteins Production for Meat and Fish Analogues

**DOI:** 10.3390/molecules28072966

**Published:** 2023-03-27

**Authors:** Malgorzata Nowacka, Magdalena Trusinska, Paulina Chraniuk, Federico Drudi, Jakub Lukasiewicz, Nam Phuong Nguyen, Adrianna Przybyszewska, Katarzyna Pobiega, Silvia Tappi, Urszula Tylewicz, Katarzyna Rybak, Artur Wiktor

**Affiliations:** 1 Department of Food Engineering and Process Management, Institute of Food Sciences, Warsaw University of Life Sciences—SGGW, Nowoursynowska 159c, 02-776 Warsaw, Poland; 2 Department of Food Biotechnology and Microbiology, Institute of Food Sciences, Warsaw University of Life Sciences—SGGW, Nowoursynowska 159c, 02-776 Warsaw, Poland; 3 Department of Agricultural and Food Sciences, University of Bologna, Piazza Goidanich 60, 47521 Cesena, Italy; 4 Interdepartmental Centre for Agri-Food Industrial Research, University of Bologna, Via Quinto Bucci 336, 47521 Cesena, Italy

**Keywords:** meat analogues, fish analogues, ingredients, health aspects, technology

## Abstract

In recent years, there have been significant developments in plant proteins production for meat and fish analogues. Some of the key developments include the use of new plant protein sources such as soy, legumes, grains, potatoes, and seaweed, as well as insect proteins, leaf proteins, mushrooms, and microbial proteins. Furthermore, to improve the technological and functional properties of plant proteins, they can be subjected to traditional and unconventional treatments such as chemical (glycosylation, deamidation, phosphorylation, and acylation), physical (pulsed electric fields, ultrasound, high hydrostatic pressure, dynamic high-pressure treatment, and cold plasma), and biological (fermentation and enzymatic modification). To obtain the high quality and the desired texture of the food product, other ingredients besides proteins, such as water, fat, flavors, binders, dyes, vitamins, minerals, and antioxidants, also have to be used. The final product can be significantly influenced by the matrix composition, variety of ingredients, and water content, with the type of ingredients playing a role in either enhancing or constraining the desired texture of the food. There are several types of technologies used for meat and fish analogues production, including extrusion, shear cell technology, spinning, 3D printing, and others. Overall, the technologies used for meat and fish analogues production are constantly evolving as new innovations are developed and existing methods are improved. These developments have led to the creation of plant-based products that have a similar texture, taste, and nutritional profile to meat and fish, making them more appealing to consumers seeking alternatives to animal-based products.

## 1. Introduction

Plant protein production for meat and fish analogues has undergone significant advancements in recent years [1]. These developments include the use of new plant protein sources such as soy, legumes, grains, potato, and seaweed, as well as insect proteins, leaf proteins, mushrooms, and microbial proteins. To obtain the desired texture and quality of the product, these proteins can be subjected to traditional and unconventional treatments such as chemical, physical, and biological. In addition to proteins, other ingredients such as water, fat, flavors, binders, dyes, vitamins, minerals, and antioxidants are also necessary [2]. Moreover, several technologies such as extrusion, shear cell technology, spinning, 3D printing, and others are used in the production of meat and fish analogues. The technologies used for the production of these goods are constantly evolving, as new innovations are developed and existing methods are improved. These developments have led to the creation of plant-based products that closely mimic the texture, taste, and nutritional profile of meat and fish, making them more appealing to consumers looking for alternatives to animal-based products [3].

Meat analogues are typically made from plant-based proteins, as well as other ingredients such as starches, fats, and flavorings, to create a product that looks and tastes like meat. Some common types of meat analogues include plant-based burgers, sausages, and meatballs. Meat analogues can be made to resemble a wide range of meats, including beef, chicken, pork, and others. Similarly, fish analogues are also made from plant-based proteins such as soy, pea, wheat, or algae, and often contain natural flavors and other ingredients to mimic the taste and texture of fish. Some common types of fish analogues include plant-based fish fillets, fish sticks, and crab cakes. Fish analogues are often fortified with omega-3 fatty acids, which are essential nutrients found in fatty fish that can be difficult to obtain in a plant-based diet [2,4].

Both fish and meat analogues can offer several benefits for those who are looking to reduce their consumption of animal products. They are typically lower in saturated fat and higher in fiber compared to their animal-based counterparts, and can be a good source of protein, vitamins, and minerals. However, it is important to choose products that are lower in sodium and additives and higher in beneficial nutrients to ensure that they are a healthy and nutritious addition to people’s diets [5,6,7]. Considering all of the above, the selected aspects related to meat and fish analogues are described below, along with health aspects and future perspectives.

## 2. Plant Sources of Proteins for Meat and Fish Analogues Production

### 2.1. Soybean Proteins

Soybeans are among the most important plant protein sources. They owe their popularity mainly to high availability, good price, and nutritional quality [8]. Soybean is known for its wide range of food products such as instant formula, protein concentrates and isolates, and textured fibers [9]. Therefore, due to their good physical properties, such as their gelatinousness, nutritional properties, low cost, and safety, they are often chosen as a meat substitute [10]. The main protein products of soybeans are soy flour (40% protein content), defatted soy flour/flakes (44–54% protein content), soy protein concentrate (SPC, 65–70% protein content), and soy protein isolates (SPI, 85–90% protein content). They are also used as a material in vegan, lactose-free products like soya milk and texturized proteins [11]. Their proteins contain all the essential amino acids (EAAs) like animal proteins but with less saturated fat and without cholesterol. Due to its beneficial effects on nutrition and health, soybean use is continuously growing [9]. They contain quite large amounts of calcium, iron, zinc, and alpha-linoleic acid, so soybeans can be a good source of them [12,13]. They also contain satisfying levels of vitamins of group B and flavonoids; also, as previously mentioned, soybean is a complete amino acids source [14]. Furthermore, the soya proteins have protein digestibility-corrected amino acid score (PDCAAS) values close to 100% (Table 1), which means that the organism uses all the amino acid that was delivered with food.

Soybeans contain between 35% and 40% protein based on their dry weight. As a part of the legume family, soybean contains mainly globulin with two major proteins: 11S globulin known as glycinin and 7S globulin known as β-conglycinin [9]. Other protein sources within soybean include oleosins, trypsin inhibitors, and enzymes such as lipoxygenase. Trypsin inhibitors cause a negative effect on human digestion, reacting with trypsin and chymotrypsin to form a stable complex [11].

Soybean proteins also contain peptides that are typically 2–20 amino-acids long and that are inactive until they become released from an intact protein parent. Their small size makes them easily absorbed into the intestines and transported to the bloodstream [9]. Despite soybean being a popular ingredient, soybean use in food production is declining because of the clean label trend. Soybeans are often genetically modified [15], as well as having an allergenic potential [4].

### 2.2. Legumes Proteins

Legumes are great protein sources, containing high amounts of EAAs, so they can successfully substitute whey proteins [19]. They are also known as pulses and are rich in protein, which can reach up to 30% of dry weight, carbohydrates, dietary fibers, minerals, and vitamins. Like other plant protein sources, they are also a source of polyunsaturated fatty acids, especially linolenic and α-linolenic acids. Legumes in general contain high levels of lysine, leucine, aspartic acid, and arginine but low levels of sulfur-containing amino acids and tryptophan. Because of that, it is necessary to combine different legumes to obtain products containing significant amounts of all EEA [20,34]. Proteins found in legumes are globulins, prolamins, albumins, and glutelins. The main proteins of legumes are globulins accounting for about 70% of total proteins, while albumins and glutelins account for 10–20% of total proteins [19]. Legumes also contain other proteins such as prolamins and vicilins, which account for 3–7% of the total protein content [20].

One of the most widely used legume proteins is pea protein. Pea protein is popular because of its high adaptability, high nutritional value, hypoallergenic nature, and functional properties, including foam stabilization and excellent emulsification ability, making them ideal for food processing. They are very often used with other plant materials because they have the ability to complement their functions [15,27,35]. Despite its advantages, pea protein is still not as widely used as soy protein in meat analogues because of the higher cost of pea protein. As a result, soy protein remains the more popular protein used in meat and fish analogues [36].

While most legumes lack some of the EAAs, the pea is a great source of methionine, cysteine, tryptophan, and threonine with significant levels of those amino acids in its globulins, while pea albumins are rich in arginine, leucine, isoleucine, and phenylalanine. Another legume of special interest is chickpea. Chickpeas have a high arginine content, which plays a role in adenosine triphosphate synthesis, calcium release, cellular proliferation, and immunity. Opposite to other legumes, chickpeas contain all EAAs in sufficient amounts, which can be seen in their high amino acid score [20,34].

However, legumes contain many anti-nutritional factors (ANF) such as chymotrypsin and trypsin inhibitors, lectin, anti-fungal peptides, and ribosome-inactivating peptides and as protein ANFs and non-protein ANFs such as alkaloids, phytic acid and some phenolic compounds like tannins and saponins. Their main negative effects are the inactivation of digestive enzymes and hypersecretion of the pancreatic fluid. Lectin can obstruct the protein digestion process by cleaving epithelial cells and can form complexes with specified sugars and proteins lowering their digestion. Protease inhibitors are resistant to pepsin and stomach acid pH. Due to their inhibitory effect on the proteolytic enzymes, they interfere with digestion. Alkaloids can cause disturbance in the central nervous system and disorders in digestion, reproductive, and immunological systems. Phytic acid reacts with other nutrients binding with them and lowers their digestion, while phenolic compounds react with positively charged proteins [19,20].

The problems faced by food technologists in the production of meat analogues using legumes are their pungent legume aftertaste or complete lack of flavor. However, in contrast to grain protein, they can be used and consumed by people suffering from gluten intolerance [37]; as well, in comparison to soy, pea is not a major allergen.

### 2.3. Grains Proteins

Grain proteins are obtained from cereals, the main three of them being gluten, zein, and rice protein [38].

Gluten is the main storage protein of wheat and is composed of hundreds of proteins with gliadin and glutenin being the main proteins. Gluten accounts for 85–90% of total protein in wheat, with other proteins being albumin and globulin accounting for 10–15% of total protein. It is known for its functional properties and contains a great amount of sulfur-containing amino acids but cannot be consumed by people with celiac disease or gluten sensitivity [38,39].

Rice protein contains a high amount of EAAs but has fewer functional properties than gluten. The amount of proteins and their digestibility are reported to be higher than in other cereals. Opposite to gluten, rice protein is hypoallergenic and can be consumed by everyone. Amino acids found in rice are albumin, globulin, glutelin, and prolamin. Bran obtained from milling rice contains 10–20% protein and has a high amount of aromatic amino acids of 9.46–11.46%. Digestibility of rice bran proteins reaches 94.6% with 0.90 PDCAAS, and rice endosperm protein digestibility reaches 90.8% with 0.63 PDCAAS. Proteins obtained from rice bran and endosperm have similar digestibility to soy protein isolate (SPI) and dairy protein ingredients. Those proteins also have similar content of EAAs (41.1–41.7 g/100 g) to SPI (41.2 g/100 g) [21,38,40].

The most commonly used protein from this group is wheat protein. Its appropriate viscoelastic properties, as well as its rheological properties, result in meat analogues with the right consistency [35]. However, this protein, together with other grain proteins, does not have a very high nutritional value and has low levels of EAAs [13].

### 2.4. Potato Proteins

Potatoes are among the most cultivated global crops that are high-protein-providing crops per area grown. The protein content of potatoes reaches 1–2% with the main protein being patatin, known also as tuberin. Patatins account for 35–40% of protein content and protease inhibitors account for 30–40% of protein content [22].

Potato proteins isolated from potato juice are reported to exhibit high solubility and foaming and emulsifying properties. These properties, as well as their high gelling capacity, make them a very good texturizer. They have the ability to gel as low as 45 degrees Celsius, which is used when gelling products with heat-sensitive ingredients [41,42].

Of most plant-based proteins, potato proteins have the best balance of essential amino acids, comparable to animal proteins, which is even better than soybean proteins [43]. Another source reports that only wheat has a slightly better ratio of well-absorbed essential amino acids, and their nutritional quality was rated as excellent, comparable to soy [13]. Their other big advantage is also that they are gluten free, so it is possible to produce gluten-free meat [15]. Using the right method, this protein transforms and forms a network structure that resembles meat [42].

### 2.5. Seaweed Proteins

In terms of potential use, seaweed protein seems to make sense because of its fishy smell and taste. Seaweed as a marine organism does not compete with other crops for land and does not require fresh water to grow [44]. Edible microalgy is classified into three groups based on the composition of photosynthetic pigments for red, brown, and green seaweed [23]. However, its nutritional quality makes seaweed a potentially sustainable food source for humans. Its protein content ranges between 5% and 30% of dry weight, and carbohydrates content ranges from 30% up to 70% depending on species [44]. Brown seaweed has a protein content of 5–15% and contains all required amino acids in the amounts suggested by the FAO. Green seaweed protein content is estimated to be 30% greater than brown seaweed. The protein content of red seaweed is estimated to be 10–40% greater than brown and green seaweed [23]. The number of proteins in red seaweed is estimated to be between 2% and 47%. All the EAAs were found in red seaweed with the amount ranging from 31.1% (g amino-acid-N/100 g protein-N) to 42.1% compared to whey 48.1%. Some red seaweed species were found to contain ovalbumin on levels greater (36.5–42.1%) than whey (38.4%) and soy proteins (34.5%) and methionine (2.7%) on slightly higher levels (1.9% and 1.7% whey and soy protein isolates, respectively) [24].

Consuming raw seaweed can decrease the amount of digested protein because of the presence of soluble fibers and polyphenols. Proteins account for 30–50% the mass of seaweed and are digestible in 75% [23].

Generally, seaweed does not produce toxins but there is a risk of accumulation of heavy metal ions such as cadmium, lead, or mercury [23]. Seaweed can also contain anti-nutritional factors, radioactive isotopes, and ammonium [24]. Seaweed is also rich in iodine, high consumption of which may lead to health hazards. The main way to reduce the toxicity of seaweed products and raw seaweed is proper preparation and knowledge of the iodine levels in seaweed [23].

### 2.6. Other Proteins

For the meat and fish analogues products, there are also other proteins used such as insect proteins, leaf proteins, mushroom proteins, and microbial proteins. However, there is ongoing research into novel plant-based protein sources, so it can be expected that new ingredients will be studied to continue advancing the field of meat and fish analogues.

One of the mentioned sources of proteins comes from insects, which has in the last years been widely studied. Insects commonly contain 9–25% protein with some species’ protein content reaching 49–85%. Studies have shown that processed insect flour has EAA values between 6% and 59% with reference protein and 73% PDCAAS. The sum of all EAAs of insect proteins is similar to soybean proteins but slightly lower than that of casein. The digestibility of insect protein was estimated to be between 76% and 98%. Insects require lower amounts of food and water, creating less waste. They do not need to use food energy to maintain body temperature. Due to their lower requirements and waste materials, the lower environmental impact seems promising [16,17,26].

Leaves can also be used as a protein source with some studies estimating the number of proteins to be between 15% and 20% dry mass. Leaf protein concentrates (LPC) were obtained in the late 2000s and are mostly used in animal feeding. Leaf protein concentrates contain all EEA with protein accounting for around 70%, with a solubility of 90% in pH above 6 [17,45]. Calderon et al. [46] obtained protein concentrate from jackfruit with 65.82 g protein/100 g. Other studies discovered that pumpkin and amaranths leaves contain 11.75% and 10.7% protein, respectively. Additionally, moringa leaf protein flour was reported to have a total amino acid of 42.76 g/16 g N and 41.42% PDCAAS [17]. Yu et al. [47] studied the impact of age on protein content in tomato leaves. The result shows that tomato leaves contain above 20% of the protein that decreases within days after sowing crops. The initial amount of protein was 27.8%, which decreased to 24.7% after 60 days. Additionally, leaves can be a source of phenolic compounds [47].

Mushrooms were observed to produce good protein and fiber content products using 3D modeling and are already in use in meat substitutes such as patties [12,17]. Proteins obtained from edible fungi can reduce levels of LDL (low-density lipoprotein) fractions of cholesterol and increase HDL (high-density lipoprotein) fraction levels. Although having less functional properties than soy protein, fungi protein can give meat-alternative products organoleptic attributes similar to meat [12]. Mushroom proteins obtained from fungi contain high amounts of protein (45% of total mass) and great amounts of EAAs accounting for 44% of total protein. Protein ingredients extracted from fungi have a better fatty acid profile than meat products and have high fiber content. Like microorganisms, they require a lower area to grow, use less water, and can be fed with wastes from other industries. Some studies show that fungi have a similar impact on the environment to chickens with a much lower area required to grow them [31]. White button mushrooms (*Agaricus bisporus*) were utilized by Keerthana et al. [48] to create sensory snacks that contained 14.95% protein and 5.37% fiber. Another study by Teng et al. [49] used *Cordyceps militaris* to produce a product high in protein and EAAs [17]. In other research, it has been shown that the combination of chickpea protein and oyster mushroom protein (*Pleurotus okoatus*) in a ratio of 2:3 can be used to produce meatless nuggets with a positive organoleptic evaluation [50]. In addition, it has been shown that the protein hydrolysates of the *Pleurotus ostraatus* mushrooms can be an important source of both bioactive and nutraceutical compounds, which, when used in plant analogues of meat and fish, can enhance the health-promoting properties of these products [51]. It was also shown that the edible mushroom protein obtained from *Phlebopus portentosus* can be used as an emulsifier in the food industry [52].

Microbial proteins can also be obtained, especially bacterial (e.g., *Methylophilus* sp. and *Methylomonas* sp.) or yeast (e.g., *Saccharomyces* sp., *Candida* sp., *Pichia* sp.) proteins. In turn, the protein from the mold *Fusarium venenatum* is used industrially. It was found that microbial biomass of bacteria, yeast, and microalgae contain up to 83% protein. The yield of protein obtained from microorganisms is 10 times higher than the protein yield from meat production. Some bacteria and yeast can produce proteins with different sources of nitrogen with low resources causing low environmental impact. As a nitrogen source, bacteria and fungi can use waste material from agro-industrial, livestock, and human activities. Microorganisms require a smaller area for protein production (0.052 m^2^/kg protein) compared with livestock (1.047–2.279 m^2^/kg protein for beef). Using different species of microorganisms can produce protein sources greater than soy and whey proteins [17,32]. To obtain a protein preparation from *Fusarium venetatum*, continuous cultures are carried out in a bioreactor with strictly controlled temperature and pH. Then, the centrifuged biomass should be subjected to heat treatment to break down the RNA, the content of which in the preparation must not exceed 1% [53].

## 3. Conventional and Unconventional Modifications of Plant Protein

The modification of proteins consists mainly of the structural alteration (secondary and tertiary rearrangement and subunit disaggregation) of the protein molecule and the subsequent modification of the main molecular properties to improve or change their techno-functionality when used as a food ingredient [1,54].

In the field of meat and fish analogues, plant proteins not only have a nutritional purpose but also play an important role in the texture and overall acceptability of the product [55]. In any event, vegetable proteins generally do not perform as well as animal proteins in terms of technological and functional properties, normally showing lower solubility and weak, unstable gels, creating poor and unstable foams and emulsions [1].

Many different modification processes have been explored, which can be classified as physical, chemical, and biological depending on the approach used. In this section, the most important modification processes for plant proteins are briefly mentioned, and more specific review articles are provided for further reading. Additionally, Table 2 describes the mechanisms of each technique.

### 3.1. Chemical Modifications

Chemical modification consists mainly of the addition, depletion, or alteration of residues from the side chain of the amino acids, thus differing from the physical methods that are only changing the spatial conformation of the protein. This is obtained using specific conditions and, often, reagents, making these processes sometimes challenging for safety and regulatory reasons [93]. Nevertheless, the main advantages of these processes are easier implementation and high efficiencies, if compared to physical modification processes [56]. The most used mechanisms for modification are deamidation, acylation, phosphorylation, and glycosylation, the latter being the most promising because it does not use hazardous chemical reagents.

#### 3.1.1. Glycosylation

Glycosylation (also called glycation) consists of the covalent bonding between a reducing sugar (or the reducing group of polysaccharides) and the free amino group of amino acids, resulting in N-substituted glycosilamines [56]. This reaction can be achieved by enzymatic methods [94] or through a controlled Maillard reaction. The latter is gaining more and more attention as it allows the protein to be modified without the use of hazardous solvents or reagents, obtaining a food-grade product that could be named as a clean-label product just like physically modified proteins [95]; however, there are issues to be overcome caused by the time-consuming process and the off-flavor/color that can develop because of the high temperature required [94]. An extensive list of applications for plant proteins has recently been published by [57,58].

#### 3.1.2. Deamidation

Deamidation of proteins is a reaction in which amino acids with free amides in their residues, such as asparagine (Asn) and glutamine (Gln), are converted into the corresponding charged acid (aspartic and glutamic) with the release of ammonia [56]. This application can be obtained by enzymatic or chemical processes, the latter usually being carried out by acid or alkali treatment [54,59]. In these cases, high temperatures are required to accelerate the process, and normally, organic acids are more commonly used for food-grade products. However, these processes have the disadvantages of generating numerous undesirable byproducts and hydrolyzing the proteins to some extent.

The balance between the degree of deamidation and the degree of hydrolysis during treatment is crucial to obtain a partially denatured protein that can further rearrange and obtain the desired properties [56,60]. Some applications in the field of plant proteins have been reviewed by Chen et al. [59].

#### 3.1.3. Phosphorylation

Like the modification already mentioned, phosphorylation can be achieved by enzymatic or chemical processes. In the latter case, the most commonly used reagents are sodium tripolyphosphate (STP) and sodium trimetaphosphate (STMP), both of which are used in combination with dry or wet thermal treatment to achieve the desired modification [61].

This chemical technique consists of incorporating a phosphate group into the protein chain, resulting in an increased negative charge, which is ultimately responsible for the increased hydrophilicity and solubility of the protein [62]. Some applications in the field of plant proteins have been reviewed by Hu et al. [61].

#### 3.1.4. Acylation

Acylation is a process that can be divided into acetylation or succinylation depending on the functional group added to the protein chain (acetyl or succinyl group) and, thus, on the reagent used (acetic or succinic anhydride) [96]. In both cases, the reaction involves an amino or hydroxyl group of the protein, lysine being one of the most reactive amino acids in this application. These processes are able to change the spatial conformation of the protein by adding the negative charge of the succinyl group or neutralizing the positive charge of lysine in the case of acetylation, thus affecting the solubility and functional properties [56,63]. Some applications in the field of plant proteins have been recently reviewed by Heredia-Leza et al. [63] and also by Basak and Singhal [64].

### 3.2. Physical Modifications

Physical modifications consist of using different technologies to mainly induce conformational changes in the protein structure without the addition of specific reagents [65]. Ingredients obtained by these methods may receive clean labeling, which brings them a lot of attention [93]. Nevertheless, questions about the cost-effectiveness of these processes and a deeper knowledge of the mechanism behind their principles still need to be fully addressed to make them industrially reliable [97,98]. The following section provides a brief overview of new non-thermal physical treatments.

#### 3.2.1. Pulsed Electric Fields (PEF)

Pulsed electric field devices operate with short (μs-ms) and repeated electric fields in a treatment chamber (continuous or batch) that can polarize the product without heating it. The classical application of this technique is the enhancement of mass transfer or the inactivation of microorganisms since its action is usually observed in cell membranes [99].

However, the usage of this technology for protein modification has yet to surge, with the focus so far being on enzyme inactivation rather than protein functionalization [100]; moreover, the protein modification mechanism that occurs during PEF treatment is still unclear.

From the available literature, it appears that only the secondary and tertiary structures are affected, which is probably due to the induction of protein backbone alignment by molecule polarization [67] and an electrolysis reaction that contributes to its stretching [65,68]. This phenomenon can lead to the disruption of organized conformations (such as α-helix and β-sheet), which can result in a further conformational change and subsequent unfolding of the protein [66,67]. A list of applications for proteins can be found in [65,69].

#### 3.2.2. Ultrasound (US)

Ultrasounds are sonic waves above 20 kHz that are being used for food processing as they can deliver energy through a medium, enhancing efficiencies and the rate of several processes such as extraction and microbial inactivation. In addition, the focus of research has recently shifted to the effects of modification [101].

Using this process, the main principles affecting protein structure are cavitation and shear forces, both of which are capable of disrupting hydrogen and disulfide bonds [70], as well as hydrophobic interaction, while the primary structure of the protein remains generally unchanged [71,72]. This can lead to particle size reduction because of disruption of protein–protein interaction. Moreover, a denaturation effect can appear, affecting both tertiary and secondary structures [70]. A comprehensive and updated list of the US applications for plant proteins can be found in [73,74].

#### 3.2.3. High Hydrostatic Pressure (HHP)

High hydrostatic pressure treatment consists of a batch process that delivers high pressures (up to 1000 MPa) to packed products through a liquid medium (water). This technique was first applied for microbial inactivation but currently it is receiving more attention from different fields, one of which is protein modification [75].

The mechanism behind its operation is well-understood and was recently resumed by Wang et al. [76]. It all boils down to Le Chatelier’s principle, which states that the equilibrium that determines the structure of a molecule shifts toward the smallest possible volume when pressure is applied [77].

This leads to subunits disaggregation at pressures below 100–200 MPa (quaternary structure disruption), while at pressures of 300–400 MPa, the molten state can be reached (loss of tertiary structure), and complete denaturation generally occurs at pressures above 400 MPa [65,66,76]. In addition, in this case, the primary structure of the protein remains unaltered, as this technology is able to affect only weak bounding such as hydrogen bounds, disulfide bridges, and hydrophobic interactions [76]. For a recent list of publications on plant protein, see [78].

#### 3.2.4. Dynamic High-Pressure Treatment (DHPT)

Dynamic high-pressure treatment consists of a continuous process in which a liquid is forced to pass through a narrow valve, creating high pressure and shearing forces able to reduce the size distribution of emulsions and suspensions [79]. The two main processes that fall into this category are high-pressure homogenization (HPH) and microfluidization, which differ mainly in the treatment chamber and, thus, in the operating principle [80,81].

The mechanisms thought to be responsible for protein modification are the high shear forces combined with cavitation and local temperature rise, which can lead to disaggregation of subunits (loss of quaternary structure) and also to partial loss of secondary and tertiary structures. In addition, subsequential re-aggregation of partially denatured subunits can occur [82]. For a recent list of applications on plant protein, see the review paper authored by Sahil et al. [80].

#### 3.2.5. Cold Plasma (CP)

Cold plasma consists of ionized gasses with a low temperature, obtained by the application of an electric field [102,103]. This technology has been used in the food sector for packaging decontamination and seed germination, while new applications are still being studied [104].

For protein modification, CP treatments are effective at all four levels of protein structure, making it one of the only physical techniques that can also alter amino acid structure [104]. The strong oxidative capabilities of many reactive oxygen and nitrogen species (ROS and RNS, respectively) can cause hydroxylation of aromatic rings, oxidation of thiol groups, and amidation of histidine and proline [83,105].

The effects on secondary and tertiary structures show a tendency to partial denaturation of proteins, leading to better technological performances. Nevertheless, many authors point out a deterioration of the properties when the treatment time is too long or higher energies are applied [84]. This phenomenon is often explained by excessive denaturation causing subsequent aggregation between protein molecules, which in turn is responsible for the reduced solubility and functional properties [85].

For an updated list of CP applications of protein products, refer to [86,104], while for a specific list on plant protein, see [85].

### 3.3. Biological Modifications

Two important modification processes, fermentation and enzymatic modification, fall into this category. Both rely on the action of enzymes on proteins, produced in situ in one case and selectively added in the other. Enzyme-mediated reactions can result in either protein disruption (hydrolysis) or protein cross-linking, so the process must be tailored to the specific needs of the application and the requirements of the starting matrix.

In general, the use of specific and purified enzymes provides the opportunity to achieve site-specific modification while generating minimal byproducts that can be harmful or affect product performance. In addition, enzymes require milder conditions (in terms of pH and required temperature) compared to chemical modifications and are therefore often used to overcome the main problems described for glycosylation, phosphorylation, and deamidation. However, the main disadvantage of using enzymes is the high cost of the enzyme itself and the more critical role played by the environmental conditions (salt concentration, pH, and temperature) in the reaction, which needs to be better controlled and monitored [96].

Various enzymes can be used to carry out the crosslinking reaction, with transglutaminase being the most commonly used [87,106,107], followed by laccase, tyrosinase, and peroxidase [88]. In addition, the work of Wu et al. [94] provides a comprehensive review of the current research on the use of enzymes for protein saccharide glycation.

The second modification technique using enzymes focuses on the hydrolysis of proteins, reducing their molecular weight and creating peptides with new and tailored functional properties. For this purpose, protease enzymes capable of cleaving the peptide boundaries between amino acids in the primary structure are used [89]. Trypsin, pepsin, papain, and alkalase are some of the most commonly used proteolytic enzymes. A discussion of the most recent applications for plant proteins was given by Olatunde et al. [90].

Fermentation processes can also achieve the same effects on protein obtained using enzymes, with the difference that microbial growth causes more modification of the substrate because of the high production of secondary metabolites [91]. Nevertheless, this treatment also has the potential to overcome other common problems for plant protein, namely digestibility, antinutritive factors, and off-flavors [90,92].

## 4. Other Ingredients Used in the Meat and Fish Analogues

**Figure 1:**
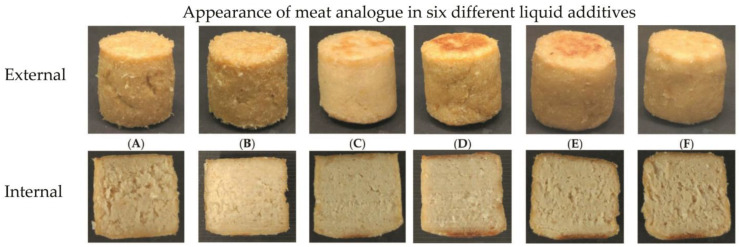
Visible appearance of meat analogues in six different liquid additives: (**A**) water; (**B**) water + soy protein isolate; (**C**) canola oil; (**D**) canola oil + lecithin; (**E**) O/W emulsion; (**F**) water + canola oil + soy protein isolate + lecithin [108].

The composition of the matrix, the variety of ingredients, and the water content have a large impact on the final product, and the types of ingredients can enhance or limit the desired texture of the food. As shown in Figure 1, different additives used in meat analogues may significantly affect visual appearance as well as the overall properties of obtained vegan products [108]. The recipe of meat and fish analogues includes water, proteins, flavors, fat, binders, dyes, vitamins, minerals, and antioxidants. Water typically makes up more than 50–80% of the total ingredients, acts as a plasticizer during the processing of meat analogues, and provides the desired juiciness [2,4].

### 4.1. Fats

Replacing animal fats with fats of vegetable and/or marine origin is in line with health recommendations (lower amounts of saturated fatty acids (SFA), higher content of mono- (MUFA) and especially poly-unsaturated (PUFA) fatty acids, etc.). Fats give meat and fish analogues the right texture and flavor. They are also responsible for combining the other ingredients. However, the use of vegetable fats in meat analogues is difficult because of the functions of fat, both as a flavor carrier and for the technological aspects of the product (mouthfeel, juiciness, texture, fat-binding properties, etc.) [109]. In meat and fish analogues, rapeseed oil, coconut oil, sunflower oil, corn oil, sesame oil, cocoa butter, and many other sources of plant and vegetable oils are most commonly used [2].

Some fish, such as salmon, are fatty fish containing more than 20% fat in their chemical composition. Its replacement in analogues is not easy, and fats that can be used in the design of analogues are constantly sought. In addition, fish is rich in omega-3 and omega-6 fatty acids, which are essential in a balanced human diet [110]. Fish analogues must contain the right composition of fatty acids to have the right nutritional value. The addition of proteins derived from sea algae, on the one hand, can give the product structure, but on the other hand, sea algae are rich in EPA and DHA acids, which have a huge impact on the development of neurons, retina, and the immune system and can also alleviate cardiovascular diseases [111].

Studies of Shahbazi et al. [112,113] demonstrated the possibility of replacing all or part of the traditionally used canola oil by surface-active biopolymers to produce a 3D-printed fibrous meat analogue with reduced fat content. Gels including dodecenyl succinylated inulin and ethyl (hydroxyethyl) cellulose offered stable structures. Thus, it seems to be possible to obtain products with a reduced amount of meat fat by the 3D-printing method and because of the obtained gels with high pseudoplasticity.

In the beef patty analogues, coconut oil and canola oil (7.75–7.81% each) were used as fatty substances. In the analogues, the content of C22:6n3, C20:4n6, C16:1, and C14:1 was significantly reduced, whereas the content of C20:5n3 (EPA), C18:2, C12:0, C14:0 (myristic acid) and C20:0 (arachidic acid) was increased compared to beef patties. Differences between the control and plant-based samples may be due to the muscle tissue of the meat, as the main fatty acids in beef are long-chain fatty acids such as C16:0, C18:0, C18:1, and linoleic acid (C18:2), while in vegetable oils they are mainly C18:3n3, C16:1, C18:0, C16:0, and C18:1 [2,114]. It was shown that in plant-based burgers containing soy-textured protein, fatty acids such as C18:0 and C16:0 were decreased in comparison to meat burgers [115].

A promising trend seems to be the acquisition of fat for meat analogues by cell culture. The use of cell-cultured fat cells (adipocytes) together with vegetable protein can therefore significantly improve the quality and consumer perception of plant-based meat without compromising sustainability. In vitro breeding may allow for the control of lipid profiles, and this leads to obtaining products that will have better nutritional parameters [116].

### 4.2. Structuring Ingredients

Giving the right texture and structure to plant analogues of meat and fish is crucial when determining the composition of new products. The texture of the fish is characterized by elasticity and a feeling of disintegration when chewed. Texture mimicry is critical to the overall quality and consumer acceptance of seafood substitutes [35]. The structure of proteins depends on their type. Proteins show important structure–function relationships in terms of emulsifying and foaming properties, flavor binding, viscosity, gelation, and texturization [4]. The main method of obtaining the appropriate structure of meat and fish analogues is the use of textured vegetable proteins (TVP) [35]. Another alternative technique to mimic texture is to bind plant proteins to polysaccharides, an example of which is alginates, which can form strong protein-capturing polysaccharide gels. The additional use of microbial transglutaminase causes the proteins to be cross-linked, creating solid gel networks [Moreno et al., 2008]. Another ingredient that enables the formation of the structure of plant analogues is konjac glucomannan, which was used in fish balls, where the correct parameters of hardness, chewiness, and gel strength were found [117]. Moreover, the use of konjac glucomannan has been shown to extend digestion and reduce the release of metabolites from fish-ball analogues, which may help control appetite and lower postprandial blood glucose levels [118]. An interesting ingredient that can be used in the production of extruded meat analogues may be defatted peanut flour. Sensory analysis of meat snack analogues showed better acceptability of products with peanut flour than with the addition of soy protein because of the lack of an unpleasant aftertaste [119].

### 4.3. Coloring Ingredients

The color of plant-based alternative products is a crucial factor affecting food perception, attractiveness, and quality. Plant-based products with intense, typical colors similar to their animal patterns tend to attract more consumers and encourage them to make purchases. When creating substitutes for meat and fish, consideration must be given to the form of product consumption since the color of meat products changes when cooked. The proteins responsible for the characteristic color of meat, such as myoglobin, undergo chemical changes during cooking, which must be considered when designing analogues [2,114].

Analogues of animal products typically incorporate natural dyes in extract form. The study of Shahbazi et al. [113] showed that beet juice extract (0.3% *v*/*v*) could be utilized as a colorant in meat analogues. The same dye is also used in commercial meat analogues; however, it can be replaced with tomato paste, too. Other meat analogues available in the market use soy leghemoglobin. This coloring ingredient is a soy-derived compound with similar chemical and structural properties to hemoglobin and myoglobin (responsible for the color of raw meat). Furthermore, in plant-based patties that mimic red meat (beef), the incorporation of lactoferrin and red yeast rice give nice colors. However, these ingredients reduced the acceptability in terms of both taste and texture of the products obtained [114]. Additionally, pumpkin powder (3% *w*/*v*) was used to impart color to cooked chicken breast analogues, but higher L* values were observed in the meat compared to its analogues [120]. While Remya et al. [121] used paprika dye to obtain a shrimp analogue. Incorporating 2.74% anthocyanins extracted from aronia was found to enhance both the color and antioxidant properties of plant-based analogues [122].

Microbial-derived carotenoid pigments, such as astaxanthin produced by *Phaffia rhodozyma* or *Yarrowia lipolytica* yeast, can also be utilized in analogues of fish products, such as salmon analogues. Astaxanthin is a dye commonly used in salmon nutrition to provide it with a distinct color. Moreover, astaxanthin has over 500 times more antioxidant activity than vitamin E and other carotenoid pigments like lycopene, along with antimicrobial activity that may prolong the shelf life of fish [110,123,124].

### 4.4. Flavoring Ingredients

Flavors associated with meat, fish, and seafood arise from volatile and non-volatile ingredients, which vary in concentration depending on several factors such as species, organism age, environmental conditions, and product freshness. Additionally, aquatic products possess the fifth primary taste—umami. Umami taste is described as savory and intensified by certain amino acids such as glutamate, glycine, alanine, and aspartate [125]. Plant-based analogues acquire meat and fish flavors through the addition of appropriate spices or by the Maillard reaction, which by reacts sugars and amino acids into flavors [111]. Attempts are also made to isolate certain naturally occurring volatile compounds and then to combine them and add them to the final product in small amounts [2]. For example, in shrimp analogues, natural shrimp flavor was used to impart a characteristic smell to the final product [121].

### 4.5. Vitamins and Minerals

Vitamins and microelements play a crucial role in the chemical composition of plant-based alternatives to meat and fish. These nutrients can be incorporated into the product through biofortification and supplementation methods [126]. Vitamins are also present in products that are ingredients of vegan fish, such as algae, which are rich in vitamins B and E [127].

### 4.6. Polysaccharides

Polysaccharides are becoming important ingredients for use in meat and fish analogues because of their gelling and thickening functions, modification of rheological properties, and better water-binding capacity [128]. They can be divided into two groups, i.e., flours and starches, and binding ingredients, i.e., gums (gum arabic, guar gum, and xanthan gum). In addition, methylcellulose is a common ingredient used in the production of meat and fish analogues. It is a plant-based compound that is often used as a binder and emulsifier to improve the texture and mouthfeel of plant-based products. Both insoluble and soluble polysaccharides play an important role in improving the rheological properties and interactions between the protein, lipid, and water components of the processed food system. Carbohydrates have been proven to help catalyze these components and create a stable structure [2]. Some parameters, e.g., chewing, water absorption capacity, and bulk density of defatted soybean flour extrudates, can be increased by adding sodium alginate [129]. Pectin, carrageenan, guar gum, cellulose, xanthan, and carob gum commonly act as binders and fillers in plant analogues. Starch can be used as a fat-like substance. Blending protein ingredients and starch and cellulose was very effective in creating meaty and juicy aftertaste products, as well as avoiding dominant, interfering flavors from single ingredients. The addition of soy fiber (5–10%) resulted in a more directional and finer texture of meat analogues [4].

## 5. Technologies Used for Meat and Fish Analogues Production

Creating a meat-like texture, which is a fibrous structure, is regarded as one of the major challenges connected with the production of meat and seafood analogues. There is a wide range of processing techniques that have been developed for plant-based meat analogues. The processing techniques can be classified into bottom-up and top-down structuring techniques. According to the bottom-up approach, individual fibers are assembled to form the end product, while the top-down approach assumes that fibrous structures are developed by forming a biopolymer blend with the use of an external force. The bottom-up strategy includes wet spinning and electrospinning, whereas the top-down strategy includes extrusion, shear cell, 3D printing, freeze structuring, and the blending of hydrocolloids. Various processing conditions can result in diverse structures such as fibrous, layered, or homogeneous ones. Owing to robustness and large-scale production ability, the two most common texturization techniques are extrusion and the shear cell methodology [3].

### 5.1. Extrusion

**Figure 2:**
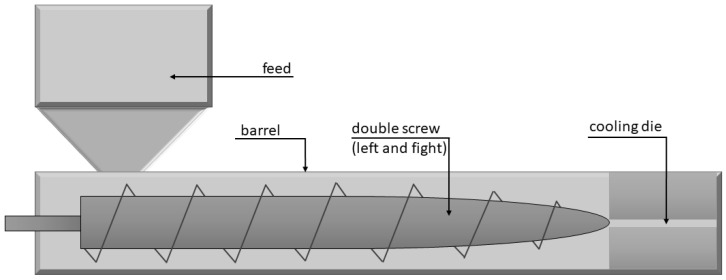
Scheme of twin-screw extruder (elaborated on the basis of Kazir e Livney [125]).

Despite many technological advances, extrusion remains the most common technique to transform amorphous plant-based proteins into fibrillar structures [130]. The principle of the method is to subject a food mixture containing proteins to hydration, high temperature, high pressure, and mechanical interactions. Extruders consist of three main parts: an electrical motor to drive the screw, one or two screws embedded within a temperature-controlled chamber, and a shaping die [43]. As the materials move through these sections, the mass is first intensively mixed and hydrated, and then, the proteins are denatured and aggregated [131]. For the production of plant-based analogues, a co-rotating twin-screw extruder is employed (Figure 2), providing a high mixing efficiency and a high output [127]. Extrudates are made in three main steps: (1) pre-conditioning of raw materials; (2) extrusion—mixing, hydration, classification, and cooking using high temperature and pressure; and (3) cooling and final processing (for example, cutting into desired pieces) [132]. Mixture composition, screw and matrix construction, extrusion parameters such as processing temperature and pressure, and screw speed will affect the final product texture [133].

There are two types of extrusion, low-moisture extrusion and high-moisture extrusion, resulting in products with different properties. Protein ingredients in low-moisture extrusion are processed with low water content (below 30%) [134]. Sudden changes in pressure and temperature at the exit of the nozzle cause the mass to expand by rapid evaporation of superheated water. The construction of the matrix determines the shape of the final product: pieces, slices, and granules [135]. Low-moisture extrusion is primarily used to produce a textured vegetable protein (TVP). Products of dry extrusion are characterized by a porous, spongy structure, high water-binding capacity, and low water and fat content—requiring hydration prior to further processing [136]. TVP has been used as a filler for meat products and as a meat substitute in the production of plant-based versions of minced meat, burgers, or sauces [135,137]. However, dry-extruded meat analogues have limited acceptance because of an unsatisfactory mouthfeel [36].

High-moisture extrusion (above 40% of water) enables the production of food proteins with characteristic fibrous structures, mimicking muscle-like texture [134]. High-moisture extrusion consists of processing protein ingredients in conditions of increased humidity. The laminar mass flow structure within the cooling section of an elongated matrix allows for the formation of a fibrous and anisotropic structure because of the crosslinking of the proteins [134,138,139]. The cooling die has a rectangular slit shape, but new cylindrical forms that increase the product output are emerging [Buhler 2020]. Products of wet extrusion have a similar moisture level to meat and have a mouthfeel similar to cooked meat [134].

The process allows for more complex formulations, and it is not necessary to use ingredients with high solubility, making it a more cost-effective technology [36]. Material suitability for extrusion is primarily determined by the ratio of soluble to insoluble components, which affects the crosslinking process of proteins [130]. The most commonly used building proteins used in high-moisture extrusion include soy protein, pea protein, and wheat proteins, but other plant proteins have also been investigated. Therefore, there is a great perspective for further expansion of extrusion usefulness in meat and fish analogue production [127,129,140].

### 5.2. Shear Cell Technology

In contrast to a not-well-defined extrusion process, a technology to produce fibrous products based on well-defined and constant shear flow deformation was introduced [141]. Shear cell technology is based on the concept of flow-induced structuring of a sheared material. The main advantage of shear cell technology compared to extrusion technologies is that shear conditions (shear rate and temperature) can be adjusted to create different product structures [142].

**Figure 3:**
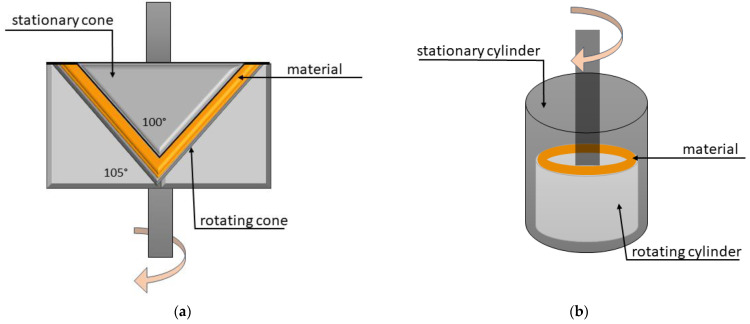
Schemes of cone-in-cone shear cell (**a**) and couette cell (**b**) devices (elaborated on the basis of Dekkers et al. [148]).

On the basis of rheometers, two shear cell designs were developed: a conical (cone-in-cone) (Figure 3a) or a couette (cylinder-in-cylinder) (Figure 3b) in which intensive shear can be generated [141,143,144]. The cone-in-cone device consists of a heated rotatory lower cone and a stationary upper cone that is lowered to obtain a sealed gap where the materials are sheared [145]. The cylinder-in-cylinder device consists of a heated stationary outer cylinder and a heated rotatory inner cylinder—the cylinders are concentric and the materials are sheared in the annulus [142]. Both devices rely on shearing under well-defined shear conditions. The sample has to be pre-mixed before being inserted into the shearing zone. The protein blend is denatured by heat and the fibrous structure is formed by the velocity profile caused by the velocity difference across the gap between the rotatory and stationary elements [142].

The final product depends on the mixture composition and the processing conditions. The fibrous structure desirable for meat and fish analogues is obtained using plant protein blends (soy protein concentrate, soy protein isolate, and wheat gluten and pectin) and calcium caseinate [128,141,145,146]. The structures produced with calcium caseinate showed anisotropy on a nanoscale, while the plant-based material showed anisotropy on a microscale [142]. Processing at a higher temperature gives a strong and layered product in comparison to lower temperatures resulting in a weak and non-fibrous product [147]. The shear cell enabled the production of meat and fish analogues with an increased thickness, more closely resembling whole cuts [142]. Since shear cell technology is a batch process, the production capacity is still limited.

### 5.3. Spinning

Bottom-up techniques of plant protein texturing include wet spinning and electrospinning. The fiber formation in these techniques occurs from protein solutions [149].

**Figure 4:**
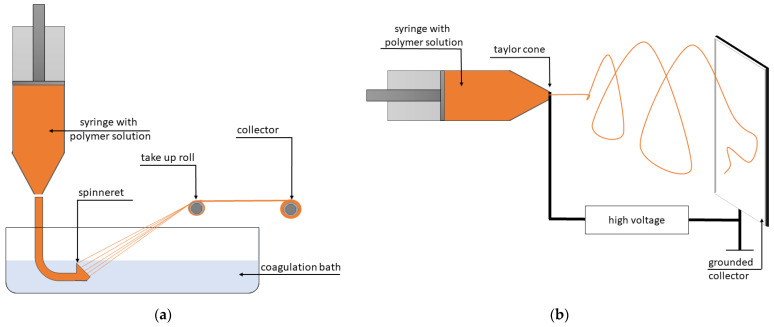
Schemes of wet spinning (**a**) and electrospinning (**b**) processes (elaborated on the basis of Kyriakopoulou et al. [55]).

In the case of wet spinning, an alkaline protein solution is extruded through a spinneret and then immersed into an acid coagulating bath containing a non-solvent for the protein aiming at precipitation and solidification of the extruded protein phase (Figure 4a). The thickness of the resulting filaments is approximately 20 μm. Wet spinning of proteins for the application of meat analogues was patented in 1954 by Boyer [15,150]. After forming, the fibers are gathered, physically or chemically treated, washed, and dried. The physical or chemical treatment aims at the improvement of the properties such as mechanical strength and molecular orientation, and the washing step aims to remove the solvents or chemicals residual on the fibers [151]. Compared to electrospinning, the fibers produced by wet spinning are characterized by larger diameter and high stiffness and strength because of a high level of molecular alignment [152]. Moreover, wet spinning enables the adjustment of the solution properties to create fibers with desirable features, as the fibers form in a liquid environment and a stable jet is not required (unlike electrospinning, in which fibers form by the solvent evaporation in air) [153]. For instance, the solidification mechanism determines the type of formed structure (fibers, capillary-filled gels, or fiber-filled gels). Fibers are obtained in the case of the solidification of the dispersed phase and the wash-away of the continuous phase; capillary-filled gels are obtained when the continuous phase is solidified and the dispersed phase stays liquid, and fiber-filled gels are obtained in the case of the solidification of both the dispersed and the continuous phases [154]. However, to conduct wet spinning, pure proteins, low pH, and high concentrations of salt and chemical additives are required [15]. Additionally, owing to the use of a significant amount of chemical reagents, this technique leads to the production of large amounts of waste (wastewater streams from coagulation and washing steps), which significantly limits its use [149].

In terms of the sustainability of food production, electrospinning is a more appropriate technique [149], which uses the electric potential relative to a grounding electrode to push biopolymer solutions through a spinneret or a hollow needle. An electrical charge accumulates on the surface of the droplets, which causes surface instability. As a result, the tiny jet coming out of the nozzle, in the form of a Tylor cone, extends into a very thin fiber (≈100 nm), and the solvent rapidly evaporates (Figure 4b) [15,125,130,149]. The three-stage mechanism of electrospinning consisting of jet initiation, elongation, and solidification into fibers was explained in detail by Osanloo et al. [155]. The electrospinning process is affected by many parameters that can be classified into three groups, i.e., polymer properties (type, molecular weight, structure, and concentration), solvent properties (viscosity, surface tension, and electrical conductivity) and ambient parameters (temperature and relative humidity) [156].

Food-grade electrospinning is mainly applied to nanofibers that can be used as carriers or delivery systems for bioactive components, such as polyphenols and probiotics. It has been reported also for some animal-based proteins: whey, collagen, egg, and gelatin [130]. Although electrospinning has been rarely applied for plant proteins, it can also be used to produce fibers for meat analogues, which was confirmed by Nieuwland et al. [157]. For electrospinning to occur and create an entangled network, the polymers need to be highly soluble, able to entangle, and the concentration should be such that there is enough overlap between the polymers [157]. Proteins are difficult to electrospin. It is feasible under the conditions that proteins are well-soluble and behave like a random coil instead of globulins. In a globular conformation, proteins may not sufficiently overlap, leading to fewer interactions with each other and insufficient entanglements for a fiber to form. Aiming at overcoming this problem, higher concentrations of protein may be used, without exceeding the maximal solubility. Plant-based proteins usually do not meet the necessary conditions of electrospinning, as in the native state they are globular, while denatured proteins form mostly insoluble aggregates. Prior to the electrospinning, plant-based proteins should be unfolded, but insoluble aggregates should not be formed, but this is difficult to obtain at once [125,130,157]. An exception is zein, owing to its amphiphilic polymeric nature [15,158]. Knowing this fact, Nieuwland et al. [157] reported that zein can act as a carrier for globular proteins, e.g., soy protein [157]. Another good solution is mixing plant proteins such as pea or soy protein with spinnable polymers such as cellulose or maltodextrin [159].

Changing conditions of electrospinning (e.g., primarily voltage, flow rate, and tip-to-collector distance) can determine the properties of plant-protein-based electrospun fibers [160,161]. Mattice et al. [162] analyzed the possibility of modifying the electrospinning parameters to create zein fibers with uniform widths and at the same time minimize the consumption of ethanol. The authors obtained tiny individual fibers; however, a very low throughput was reported, which results in problems with electrospinning with efficiency [162].

### 5.4. 3D Printing

**Figure 5:**
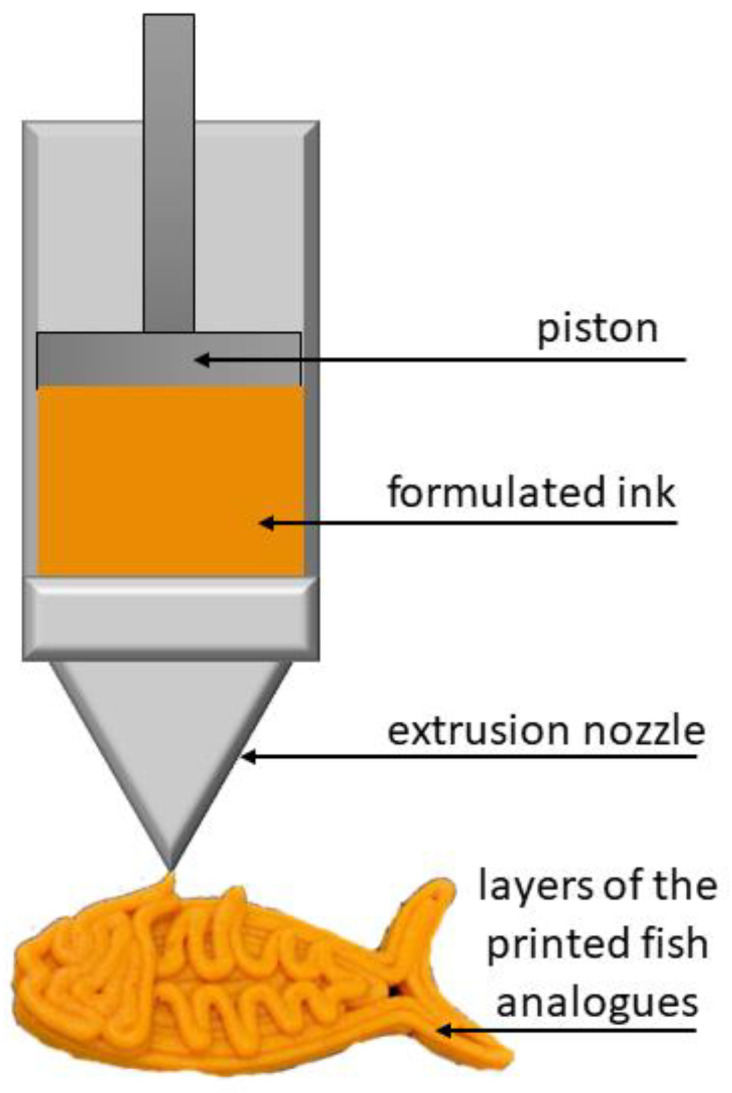
Scheme of an extrusion-based 3D food-printing process (elaborated on the basis of Godoi et al. [165]).

The 3D food-printing technique, which is also known as additive manufacturing (AM), involves a layer-by-layer deposition of materials (inks) to form 3D complex intricate structures. Depending on the fabrication principle, different techniques can be applied for food design, i.e., extrusion, sintering, ink-jet printing, and bioprinting [27,163]. The production of plant-based meat analogues most often uses extrusion-based 3D printing, which is based on the extrusion of a paste through a fine nozzle to build multilayer blocks formed layer by layer in a manner that mimics muscle fibers (Figure 5) [149]. The print is based on a pre-designed digital template. Optimized printing variables are chosen according to the required structure of the printed model. It is essential to optimize line distance, writing speed, laser power, number of layers, shape and layer thickness, printing temperatures, and cooling rates. Design and process parameters, like nozzle tip diameter, deposition rate, nozzle depth height, suck-back time, push-back time, the intensity of radiation, the temperature of hot air, and the air gap between layers are also crucial. For example, a smaller diameter nozzle makes it possible to receive a fine fibrous structure with small diameter fibers, resembling the fish-meat fibers. In turn, high printing speed and low nozzle height worsen the deposition of the printed paste, hence reducing printing precision, and as a result, a product has poor mechanical strength [163,164].

The paste, which is made of plant proteins and other components such as water, fat, polysaccharides, etc., has to have a high viscosity to obtain the desired structure of the final product. Furthermore, it should be homogenous and have appropriate printability, which refers to the physical and chemical properties, ensuring its fluidity out of the nozzle and the ability to maintain and quickly harden the 3D structure post-deposition. The chemical composition of the paste affects also the physical and mechanical properties of the final product. For instance, the molecular interactions of carbohydrates with proteins may facilitate the formation of a fibrous structure. On the other hand, too many carbohydrates may cause such an increase in viscosity, which impairs printing flowability. As fish-meat products are eaten raw, or after cooking, the printed 3D product should be durable and resistant to thermal cooking processes post-deposition [125,164,165].

One main advantage of extrusion-based 3D printing of meat analogues is the structural diversification of products [166]. Moreover, it is easy to modify the formulation to change texture, flavor, color, and nutrition [167]. Other benefits include small production space and ease of operation [166]. Environmental friendliness and technology sustainability also argue for this technique. The usage of fewer raw materials and lower energy demand link to a reduction in the ecological footprint. 3D food printing enables the merging of many steps of conventional processing. Additionally, foods can be printed on demand, which can decrease the generation of food waste [163,168]. However, because 3D-printed plant-based meat analogues are still in the initial stage, they face many challenges. Their major drawbacks are the time-consuming process, low productivity, and limited precision. Furthermore, the low extrusion force of extrusion-based 3D printers makes the hard inks of plant-based meat analogue difficult to extrude. In addition, extrusion-based 3D printing requires suitable printability, and there are limited selectable materials that can be directly printed using extruded 3D food printers and have good printability. Food materials usually need to be processed or supplemented with other ingredients before printing to modify the fluidity and gel properties of raw materials and improve the stability of the printed products [166].

### 5.5. Other Technologies

Freeze structuring (or freeze alignment) is another technique that enables the formation of a fibrous structure. During this process, plant-based proteins are blended with other components until a uniform emulsion is obtained. Then, the ice crystals are removed without melting to develop a porous microstructure with a highly connected sheet-like protein similar to that of the original meat texture [3,130]. The heat removal from a well-mixed protein solution causes the formation of an isotropic structure. However, if the heat is removed in one direction without mixing, it results in the alignment of the ice crystal needles producing anisotropic structures. Afterward, the frozen product is dried without melting the ice crystals, for example by lyophilization, to get a porous microstructure with sheet-like parallel orientation of the proteins. The aligned sheets are connected, forming a fibrous structure. Obtaining fibrous products is achievable if the proteins have relatively good solubility prior to freezing, and during the freezing process, these proteins become insoluble [8,130]. Additionally, the structure obtained by freeze structuring is determined by the plant protein source and its properties (water-holding capacity, solubility, and gelation), and also the freezing conditions (duration, temperature, the rate of freezing, pH, the solids content of the material, surface effects, heat exchange effects, degree of confinement, and pressure effects) [15,169]. The freeze-structuring technique has found its application in developing fibrous structures of meat analogues. An anisotropic layered structure similar to the structure of meat was created by Chantanuson et al. [170]. The authors formed multilayered structures from the combination of soy protein isolates. They reported that an addition of 10% soybean flour in the formulation caused the formation of dense fiber layers, stacked with a rather porous and anisotropic structure, and a tensile strength comparable to meat [170]. Furthermore, plant-based nuggets were prepared with the use of freeze structuring by Yuliarti et al. [35]. The researchers applied the texturization of a mixture of pea protein and wheat protein in a ratio of 3:1 [35].

Fibrous products can be obtained also by mixing protein with hydrocolloids that precipitate with multivalent cations. After mixing, the fibrous products are washed and the excess water is removed by pressing, yielding dry matter contents between 40 and 60 wt.% [130]. One of the major potential advantages of this technique is having no requirement for expensive and energy-intensive structuring equipment [171]. For this reason, the process is well-scalable and yields products with some degree of structure. On the other hand, it is relatively intensive in its use of resources and, in spite of the initial ordering in the shear direction, the further stages destroy this large range ordering, which limits the use of this approach to minced meat products, such as burgers and schnitzels. In this process, various combinations of proteins, hydrocolloids, and multivalent cations can be used. In 2005, a product based on casein and alginate was introduced into the market under the brand name Valess. A similar way of processing may be applied for plant proteins such as soy, rice, maize, and lupine [130]. Zhang et al. [171] reported the formulation of the scallop analogue on the basis of pea protein and high methoxy citrus pectin as the protein and polysaccharide, respectively. Heat-denatured pea protein (10%, *w*/*w*) and pectin (0–1%, *w*/*w*) were mixed to produce phase-separated biopolymer blends. Then, the blends were subjected to mild shearing (350 rpm) to obtain fibrous structures, which were then placed in molds and set by gelling the pea proteins using transglutaminase (2%, *w*/*w*) [171].

## 6. The Meat and Fish Analogues’ Products and Health Aspects

Meat and fish are primarily consumed for their high-quality protein content. If such products are completely replaced with analogues, the latter should provide comparable nutritional value. To be viable alternatives to meat or fish, analogues must have similar overall composition and proportion as the meat of fish, as well as a similar PDCAAS. Soy is a popular option for meat and fish analogues as it has a high PDCAAS score, but soy is a major allergen [4]. There is a possibility to use other protein ingredients with complementary amino acid compositions, such as combinations of cereal and legume proteins [55].

Meat and fish analogues can help create a more balanced diet with additional health benefits. Such analogues are typically lower in saturated fat compared to their animal-based counterparts, which can help to reduce the risk of heart disease and other chronic health conditions [12]. In addition, plant-based meat and fish analogues are often higher in fiber, as well as in the health-promoting phytochemicals found in plant-based products, which can help to promote digestive health and lower the risk of certain diseases. Additionally, many meat and fish analogues are fortified with vitamins and minerals such as iron, vitamin B_12_, and omega-3 fatty acids, which can be beneficial for people who follow a vegetarian or vegan diet [172].

However, there are also some drawbacks when it comes to meat and fish analogues, as well as to plant-based compounds that contain antinutritional factors (alkaloids, polysaccharides, protease inhibitors, saponins, phytates, etc.) [173]. Generally, all plant-based products had longer ingredient lists than animal meat products [172]. They contain usually high amounts of sodium, which can increase the risk of high blood pressure and other health problems. Often, meat and fish analogues contain some food additives such as preservatives, colorings, and flavorings, as well as texturing agents, e.g., maltodextrin [12]. The effects of food additives and preservatives in vegan products on human health can vary depending on the type and amount of additive used. While some additives are considered safe and have been approved for use by regulatory bodies [174], others may pose health risks if consumed in excessive amounts. Some individuals may also be sensitive or allergic to certain additives, which can lead to adverse reactions [175].

Overall, meat and fish analogues can be a healthy and nutritious addition to a plant-based diet [55]. However, the nutritional content of meat and fish analogues can vary widely depending on the brand and type of product. It is important to choose products that are lower in saturated fat, and lower in sodium and additives, as well as higher in fiber and other beneficial nutrients. Nevertheless, most of the meat and fish substitutes cannot be considered as a complete replacement for animal-origin products in terms of nutritional value [172].

## 7. Conclusions and Future Prospective

The future prospects for meat and fish analogues are promising, as the demand for plant-based alternatives to animal-based products is increasing for various reasons, including health, environmental, and ethical concerns. The development of new protein sources and improvement in technology and processing methods are expected to result in the production of more plant-based meat and fish analogues that closely resemble the taste, texture, and nutritional profiles of animal-based products. The use of more sustainable and environmentally friendly ingredients and production methods is also likely to be an important consideration in the development of these products. Additionally, further research into the health benefits of consuming plant-based meat and fish analogues, as well as the impact on human nutrition, is expected to drive innovation and development in this field. Overall, the future of meat and fish analogues looks promising, as plant-based options continue to gain popularity, and advancements are made in production technology and ingredient sourcing.

## Figures and Tables

**Table 1:** Protein digestibility-corrected amino acid score (PDCAAS) and digestibility for different sources of plant proteins.

Source of Protein	Protein Content [% Dry Weight]	PDCAAS [%]	Digestibility [%]	References
Soy	35–40	91–100	71–86	[16,17,18]
Legumes	<30	20–60	70–89	[17,18,19,20]
Grains	10–20	63–90	90.8–94.6	[21]
Potato	1–2	67–81	100	[18,22]
Seaweed	2–47	96	75	[23,24,25]
Insects	9–85	73	76–98	[17,26,27]
Leaves	15–20	41–91	75–80	[17,28]
Mushrooms	19–37	36–70	43–80.5	[29,30]
Microbial	-	91	59–78	[25,31,32,33]

**Table 2:** The mechanism of the different methods used for protein modifications.

	Technique	Mechanism	References
Chemical	Glycosylation	Covalent binding of reducing sugars to free amino groups	[56,57,58]
	Deamidation	Conversion of amino acids with free amides to the corresponding charged acids	[56,59,60]
	Phosphorylation	Incorporation of phosphate groups into the protein chain	[56,61,62]
	Acylation	Addition of acetyl o succinyl groups	[56,63,64]
Physical	Pulsed Electric Fields (PEF)	Unclear, probably molecule polarization and electrolysis	[65,66,67,68,69]
	Ultrasound (US)	Cavitation and high shear forces disrupting hydrogen and disulfide bonds	[65,66,70,71,72,73,74]
	High Hydrostatic Pressure (HHP)	Molecular stretching toward smaller volumes	[65,66,75,76,77,78]
	Dynamic High-Pressure Treatment (DHPT)	Cavitation and high shear forces	[79,80,81,82]
	Cold Plasma (CP)	Oxidation due to exposure to ROS/RNS	[65,66,83,84,85,86]
Biological	Fermentation	Enzyme produced by microorganisms reduces the anti-nutritional factors	[87,88,89,90]
	Enzymatic	Enzyme mediated crosslinking or hydrolysis	[90,91,92]

## Data Availability

Not applicable.
